# Incidental thyroid cancer and overdiagnosis: response to Drs Tsybrovskyy, Sobrinho-Simões, and Tallini

**DOI:** 10.1530/ETJ-24-0296

**Published:** 2024-10-14

**Authors:** Inês Cosme, Ana Figueiredo, Sara Pinheiro, Valeriano Leite

**Affiliations:** 1 Department of Endocrinology, Unidade Local de Saúde Santa Maria, Lisbon, Portugal; 2 Department of Endocrinology, Instituto Português de Oncologia de Lisboa Francisco Gentil, Lisbon, Portugal

Dear Editor,

Drs Tsybrovskyy, Sobrinho-Simões, and Tallini (1) argue that, when we conclude that the incidental detection of tumors larger than 2 cm suggests that overdiagnosis is the most likely cause for the rise in the incidence of thyroid cancer (TC), we are equating carcinomas found incidentally with ‘overdiagnosis’. According to Tsybrovskyy *et al*., the term ‘overdiagnosis’ can only be attributed to a condition that is both incidental and harmless, which would not be the case in our study since a significant proportion (36%) of incidental thyroid carcinomas (ITC) were larger than 2 cm in size, and only 61.5% of the patients were completely disease free at the 5-year follow-up. We think that Tsybrovskyy *et al*. have misinterpreted our writing since our conclusion, which was clearly stated in the manuscript, is that because a significant proportion of ITC patients had tumor diameters above 20 mm, overdiagnosis, and not environmental or lifestyle risk factors, is the most likely cause for the rise in the incidence of TC that is occurring worldwide. This is so because the rising incidence of TCs of all sizes and stages at diagnosis has been an argument used by others to consider that overdiagnosis alone is unlikely to account entirely for the increased TC incidence (2).

It is important to note that in our paper 30.3% of patients (3) with ITC had an indeterminate response at the 5-year outcome, a condition that only progresses to structural disease in 15–20% of the cases (4) and only 8.2% had disease persistence, either biochemical or structural, at the end of follow-up. Therefore, the prognosis of ITC does not seem as dismal as Tsybrovskyy *et al*. seem to imply. Nevertheless, in our understanding, it seems difficult to establish a cutoff on tumor size that clearly discriminates between harmful and harmless incidental neoplasias.

Tsybrovskyy *et al*. also suggest that some cases of PTC in our series might represent a non-invasive encapsulated follicular variant of PTC (NIFTP) instead of PTC since a histopathological review was not performed. As mentioned in the ‘Materials and methods’ section of the manuscript, patients included in this series were operated on in 2017 and 2018 at a time when NIFTP diagnostic criteria were already followed by our pathologists. Therefore, we do not think that ‘overdefinition’ was an issue in our paper.

## Declaration of interest

The authors declare that there is no conflict of interest that could be perceived as prejudicing the impartiality of the study reported.

## Funding

This work did not receive any specific grant from any funding agency in the public, commercial, or not-for-profit sector.
